# Task shifting and task sharing in the health sector in sub-Saharan Africa: evidence, success indicators, challenges, and opportunities

**DOI:** 10.11604/pamj.2023.46.11.40984

**Published:** 2023-09-11

**Authors:** Brenda Mbouamba Yankam, Oluwafemi Adeagbo, Hubert Amu, Robert Kokou Dowou, Beryl Gillian Mbouamba Nyamen, Samuel Chinonso Ubechu, Pascal Georges Félix, Ngwayu Claude Nkfusai, Oluwaseun Badru, Luchuo Engelbert Bain

**Affiliations:** 1 Department of Statistics, Faculty of Physical Sciences, University of Nigeria, Nsukka, Nigeria; 2 Department of Community and Behavioral Health, College of Public Health, University of Iowa, Iowa, USA; 3 Department of Sociology, Faculty of Humanities, University of Johannesburg, Johannesburg, Auckland Park, South Africa; 4 Department of Population and Behavioral Sciences, School of Public Health, University of Health and Allied Sciences, Hohoe, Ghana; 5 Department of Epidemiology and Biostatistics, Fred N. Binka School of Public Health, University of Health and Allied Science, Hohoe, Ghana; 6 Department of Economics, Faculty of Economics and Management Sciences, University of Bamenda, Bamenda, Cameroon; 7 Yale School of Public Health, Yale University, New Haven, Connecticut, United States of America; 8 Intellectual Consortium LLC, Tampa, Florida, United States of America; 9 School of Nursing and Public Health, University of KwaZulu-Natal, Durban, South Africa; 10 Usmanu Danfodiyo University Teaching Hospital, Sokoto, Nigeria; 11 Institute of Human Virology, Abuja, Nigeria; 12 International Development Research Centre, IDRC, Ottawa, Canada

**Keywords:** Task shifting, task sharing, evidence, challenges, opportunities, health systems, sub-Saharan Africa

## Abstract

This review explores task shifting and task sharing in sub-Saharan African healthcare to address workforce shortages and cost-effectiveness. Task shifting allocates tasks logically, while task sharing involves more workers taking on specific duties. Challenges include supply chain issues, pay inadequacy, and weak supervision. Guidelines and success measures are lacking. Initiating these practices requires evaluating factors and ensuring sustainability. Task shifting saves costs but needs training and support. Task sharing boosts efficiency, enabling skilled clinicians to contribute effectively. To advance task shifting and sharing in the region, further research is needed to scale up effective initiatives. Clear success indicators, monitoring, evaluation, and learning plans, along with exploration of sustainability and appropriateness dimensions, are crucial elements to consider.

## ESSAY

**Defining task shifting and task sharing:** there is an ongoing global dearth of skilled healthcare professionals, and more than four million people are needed in the global health workforce, which is a critical shortage in many parts of sub-Saharan Africa (SSA), Asia, and the Americas [1-4]. Task shifting and task sharing were developed as an ideal solution to the shortage of skilled healthcare workers [5]. Task shifting is the practice of giving specific tasks to healthcare workers who have not typically done them as part of their scope of practice [4,6]. It entails assigning tasks to healthcare workers who are more readily accessible, have less skills or narrowly tailored training, and have fewer qualifications [6,7]. Task shifting, according to the World Health Organization (WHO), is “a process whereby specific tasks are moved, where appropriate, to less-skilled healthcare workers in order to make more efficient use of the available human resources for health and by rapidly increasing capacity while training and retention programs are expanded” [4,6].

In 2010, the Institute of Medicine (IOM) officially introduced task sharing in the scientific literature to develop African capacity for HIV/AIDS prevention, treatment, and care [4,8]. Task sharing is described by WHO [6] as “an increase in the number of healthcare workers who can provide appropriate health services”. The idea illustrates how teams of healthcare professionals from various cadres work together to complete the full clinical task. The capacity to carry out specific tasks is granted to extra cadres rather than being transferred from one cadre to another. As stated by Robertson [9], task sharing involves collaborating teams of experts and less-qualified cadres who share clinical responsibility and rely on iterative communication and training to keep high-quality results. It can be a critical strategy in many settings to address the shortage of higher-level providers. Task sharing can deliver services more effectively and affordably, even in health systems with ample resources [10]. Despite differences in sharing models between countries, published evidence shows that having many healthcare workers to provide essential care can greatly increase access to healthcare services [11].

Task shifting and sharing aim to efficiently use human resources to improve the health of extremely vulnerable populations and increase cost-effectiveness. These strategies stress that all health worker cadres must receive training and ongoing educational support to accomplish the required tasks [6,10,12,13].

**Effectiveness of task shifting and sharing - evidence from the literature:** task shifting and sharing have been successfully used to improve health in various settings and times, including the COVID-19 pandemic, to address global health workforce shortages and inadequate access to care for critical health issues [4,14-17]. Some studies have shown that task sharing and shifting are efficient and effective methods for assisting healthcare workers in completing duties not previously within their purview and increasing human resource for health (HRH) and quality outcomes [14-18]. For instance, Amani *et al*. [17] implemented task shifting in Central Africa Republic in 2022 to increase COVID-19 vaccination uptake and observed that the administration of injectable COVID-19 vaccines by CHWs were highly effective and widely accepted as vaccination coverage of COVID-19 tripled from 9% in January 2022 to 29% by August 2022. Gibson *et al*. [16] conducted a task-shifting study in which CHW cadres dispensed vaccines in 20 countries with established community health worker (CHW) programs and recorded improved access to immunization rates in zero-dose communities.

Furthermore, the scoping review by Okoroafor and Christmals [18] revealed that many African countries had received substantive investments from donors and partners in recent years to shift and share task in the health sector ensuring access to health services which prove effective. Dawson *et al*. [19], indicated that delegating and sharing duties might improve patient outcomes or performance while also increasing access to maternal and reproductive health (MRH) services. Additionally, if ongoing investments are made in the healthcare system, collaboration with community members and healthcare professionals at all levels has the potential to implement MRH interventions successfully [19].

Moreover, Farley *et al*. [8] indicated that task sharing can promote decentralization in South Africa without negatively affecting patient outcomes. It may be possible to make the best use of human resources and increase access to treatment with models that permit sharing duties for multidrug-resistant tuberculosis (MDR-TB).

Additionally, some studies have shown the efficacy of task-sharing interventions for controlling blood pressure in low-and-middle-imcome countries (LMICs) and found that including non-physician healthcare workers in lowering blood pressure is a useful option in LMICs, leading to task-sharing intervention's efficacy [20]. Enhancing efficacy must be evaluated considering the surrounding conditions and the need to consider the quality, acceptability, and feasibility of care [5,21].

### Theoretical frameworks/conceptual models on responsible task shifting and sharing in SSA

***The WHO global recommendations and guidelines on task shifting and sharing:*** the World Health Organization (WHO) has devised global recommendations and guidelines on task shifting and sharing to support and guide the expansion of healthcare initiatives and workforce organization in countries [7,22]. The framework aims to establish conditions for safe, equitable, efficient, effective, and sustainable task shifting. Implementation of these guidelines aims to achieve key outcomes such as strengthening healthcare workforce capacity, improving access to services, enhancing program implementation, and bolstering health systems for high-quality treatments. The guidelines propose that countries facing healthcare workforce shortages consider adopting and enhancing task-shifting strategies in collaboration with stakeholders, alongside efforts to increase qualified healthcare workers. The importance of involving stakeholders from the outset is highlighted to ensure shared responsibility, active partnership, and alignment with national needs. The guidelines recommend a consistent national agenda for healthcare services across public and private sectors, defining roles of diverse healthcare providers and updating analyses with demographic and quality data. Task shifting and sharing should be tailored to individual country requirements, as underscored by the guidelines [22].

**Concepts and Opportunities to Advance Task Shifting and Sharing (COATS) framework:** the COATS framework is a comprehensive and adaptable model that can be used to improve task shifting and sharing in creating policy, programs, assessment, and analysis. The COATS framework consists of three elements, and this includes:

***Definition and purpose of task shifting and sharing programs:*** the COATS framework expands WHO's task shifting and sharing concept, effective for healthcare in resource-limited communities [22,23]. Task shifting optimizes labor by redistributing tasks, easing service bottlenecks [10]. It shifts tasks from skilled to less-trained personnel [24], often prompting broader healthcare system changes. The goal is to reduce disease burden and mortality by deploying less-trained professionals for efficient interventions [10,22]. Effective implementation of task shifting and sharing requires funding, planning, training, and education, enhancing resource-constrained health systems [24-26].

**Figure 1:**
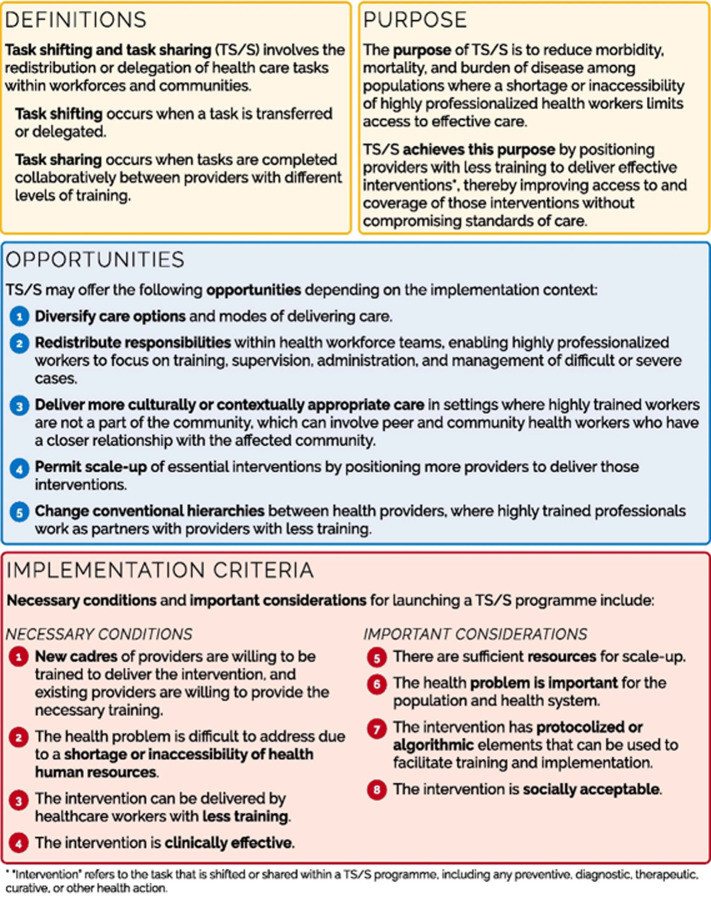
a conceptual framework for task shifting and sharing (source: Orkin *et al*. [10])

***Opportunities arising from task shifting and sharing programs:*** the COATS framework (Figure 1) outlines four task shifting and sharing opportunities to enhance healthcare systems, tailored to program context. These include diversifying care options, redistributing responsibilities to highly trained workers, culturally/contextually suitable care via peers/CHWs, and expanding interventions by altering provider hierarchies [4,10].

***Implementation criteria for task shifting and sharing programs:*** program developers, policymakers, and stakeholders can use the COATS framework's criteria to determine whether a particular situation and setting are appropriate for task shifting and sharing. “Necessary conditions” and “important considerations” constitute the criteria for putting into practice a task shifting and task-sharing program [10]. Necessary conditions that must exist for a task shifting and sharing program to succeed in achieving its goals and objectives include the characteristics of the staff that is accessible, the health issue, and the intervention. Important considerations signify ideas that might not always be relevant and will help the program succeed in some circumstances.

**Examples and outcomes of task shifting and task sharing interventions:** a summary of interventions and outcomes of task shifting and task sharing is shown in Table 1.

**Table 1:** summary of findings

Authors	Type of intervention	Outcome	Opportunities	Challenges
Gibson *et al*. [16]	Community health workers (CHWs) as vaccinators	CHWs safely administered vaccines in 20 countries, improving access to immunization services for under-reached communities.	Adequate training of CHWs will effectively provide vaccines in under-immunized communities.	CHWs faced: insufficient supply chain training, transportation for materials, heavy workload, insufficient and inconsistent pay, and insufficient supervision.
Le *et al*. [1]	Task sharing mental health interventions.	Motivation and optimism, Self-efficacy and skills.	Task sharing help improve the mental health of the patient.	Patient characteristics, organizational and structural barriers, including infrastructure and political aspects.
Indravudh *et al*. [25]	HIV self-testing approach delivered in the community.	The community-led HIVST intervention increased the number of people who recently underwent HIV testing, and there was no indication of any unintended consequences from widespread HIV anxiety or social stigma.	A change in perceived social cohesion, shared HIV concern, critical awareness, or local HIV stigma did not appear to have any indirect effects on the individual level.	A drawback of this research was the use of a cross-sectional survey to assess the outcome, potential mediators, and mediator-outcome confounders.
Ayuk *et al*. [14]	Provision of injectable contraceptives by CHWs.	CHWs who had received adequate training were able to safely administer injectable contraceptives. CHWs aid in increasing access to residents of hard-to-reach areas. 70% of clients were satisfied with CHWs as providers and DMPA as a method of contraception.	Before implementation, CHWs and their supervisors were trained to perform effectively in the community. Some women received FP for the very first time.	There was no major challenge; clients receiving DMPA were concerned about improper syringe usage and disposal outside of health facilities by CHWs.
Orkin *et al*. [10]	A conceptual framework for TS/S was developed through an online Delphi method that involves a global panel of academics with expertise in TS/S knowledge synthesis.	A more precise definition of TS/S and an overarching goal statement to direct TS/S initiatives were provided by the COATS Framework.	The COATS Framework allows TS/S programs to enhance healthcare systems.	This study did not reflect the opinions of all TS/S experts; rather, it represented the panelists' consensus. Most panellists were from LMICs, and most of their TS/S research was done in low-income populations.
Ouedraogo *et al*. [12]	Interventions for TS/S on the adoption of family planning interventions.	Formalized TS/S helped to boost healthcare utilization, particularly as CHWs offered services in the community.	Both healthcare providers and beneficiaries benefit from TS/S. TS/S assisted in freeing up time for nurses and clinical officers to perform more complex tasks, while CHWs provided counselling and short-acting FP methods.	Commodity stockouts, professional resistance, and inadequate remuneration to suit the augmented roles/responsibilities of health workers involved in TS/S were major challenges observed in this intervention.
WHO [6]	Intervention using task sharing to increase the availability of family planning and contraception.	Higher-level clinicians can use their specialized skills more frequently by delegating routine tasks to lower-level cadres. Different providers could deliver contraception safely and efficiently, increasing accessibility and availability.	Through competency-based training, healthcare workers can provide effective family planning services.	No major challenge
de Haan *et al*. [5]	A high-quality execution procedure for giving temporary ESP to CTs.	The temporary independent authority was successful in offering CTs with an ESP.	Policymakers should pay special attention to aspects related to the social setting when implementing an innovation. Policymakers should strive to understand which misconceptions may arise in the context of innovation, and then adjust the implementation strategy accordingly.	Uncertainty and lack of knowledge about the intervention created a barrier. Acceptance of new CTs is difficult and can disrupt the implementation process, resulting in unsuccessful TS and failure to achieve policymakers' goals.
Okyere *et al*. [7]	Investigating the views and perspectives of task-shifting implementers and health workers.	Proper allocation of tasks resulted in satisfactory outcomes.	TS can help overcome the effects of a lack of health personnel. Consistent modules for staff work training offer systematic learning. It gives employees the skills they need to guarantee the delivery of high-quality healthcare.	Lack of training before task assignment, improper task assignment, a lack of motivation, and health worker burnout
Martínez-González *et al*. [3]	Physician and nurse TS in primary care intervention concerning the course of the disease and nurses’ roles.	Trained nurses achieved similar outcomes to physicians in preventing heart disease, managing dyspepsia, and reducing CHD in diabetic patients.	The treatment of stroke and CHD risk in diabetic patients were helped by nurse training that used disease-specific protocols to direct nurse-led care interventions to achieve comparable efficacy to physician-led care.	Only RCTs were included in this intervention due to the lower risk of bias and ability to estimate causal effects.
Colvin *et al*. [26]	TS intervention in midwifery services.	Midwives had the skill to deliver the required task effectively.	TS is a powerful tool for addressing maternal and newborn health human resource crises, which necessitates careful planning, implementation, and oversight.	TS placed pressure on the midwifery paradigm of care despite the fact that midwives agreed or disagreed with the ideological or practical justifications for the changes.

Note: DMPA (depot-medroxyprogesterone acetate), CHWs (community health workers), FP (family planning), COATS Framework (Concepts and Opportunities to Advance Task Shifting and Task Sharing), HIVST (Human Immuno Deficiency Syndrome Self-Testing), TS/S (task shifting and task sharing), CTs (clinical technologist), ESP (expanded scope of practice), CHDs (coronary heart diseases).

**Factors that lead to effective task shifting and sharing:** task shifting and sharing are used to increase and guarantee access to vital health services by utilizing the current medical care personnel to their fullest potential. Some of the factors which lead to effective task shifting and sharing are:

***Motivation/optimism:*** effective healthcare services through task shifting and sharing require motivated workers and confident, prepared clients, fostering successful provider-client relationships and improved outcomes [1,27]. Feiring and Lie [27] identified motivation and optimism factors: beliefs about task shifting's impacts, job satisfaction, esteem, and organizational culture. Norway's 1: 4 doctor-to-nurse ratio led them to advocate task shifting as an effective HRH utilization strategy, especially for flexible team members [27]. Provider skills and self-efficacy, nurtured within task shifting interventions, are pivotal. These attributes attract clients by enhancing their perception of provider expertise. Improved skills yield better results, elevating provider self-efficacy, trust-building, and influence for meaningful change [28]. Learning about task shifting enhances readiness for new roles and responsibilities, amplifying intervention effectiveness [27]. Ultimately, the combination of motivated providers, equipped with refined skills, and confident clients fosters successful task shifting and sharing interventions.

***Organizational factors:*** these factors are an essential component in task shifting and task sharing, which, if well managed, will result in effective and efficient task distribution, as it is thought between physicians and nurses to foster teamwork by reinforcing a supportive environment [5,27]. Effective collaboration involving an existing network that connects the different healthcare workers to support the health system is an effective factor that leads to the delivery of task-shifting and task-sharing interventions successfully. For instance, the ability to send patients to specialists and other care providers was made possible by the existing provider networks. Therefore, it leads to effective task shifting and sharing when well implemented.

***Societal factors:*** they encompass socio-economic conditions, cultural norms, and historical context, can substantially hinder task sharing and shifting interventions in terms of access, involvement, and delivery [1]. Sociocultural norms pose significant challenges. Dutch medical doctors' resistance to technology adoption was linked to historical perceptions, as indicated by de Haan *et al*. [5]. Similar findings were observed in Mangochi, Malawi, by Kok *et al*. [29]. Certain medical fields, like intensive care, embraced technological changes and expanded clinical technologist roles, whereas acceptance varied among specialties, such as surgery and rheumatology [5].

**Key barriers in task shifting and sharing:** task shifting and sharing are implemented by policymakers in many countries globally. It is an effective strategy for delivering healthcare services in many countries to improve treatment costs, availability, and safety [5]. Despite recent studies reporting the safety and cost-effectiveness of task shifting and task sharing [3,7,18], some notable barriers to implementation are discussed below [1-2,5].

***Trust, responsibility, and accountability:*** these are factors affecting the success of task shifting and task sharing [2]. Colvin *et al*. [26] reported that doctors voiced anxiety about accountability and responsibility when midwives were assigned tasks with doctors. The fear frequently results from ignorance of midwifery education and practice, the impression of variation in midwife skill and experience, and ambiguous legal guidelines governing liability in these situations. Additionally, caregivers shared the same worries.

***Organizational factors:*** effective healthcare delivery and minimal inter-professional cooperation depend on each cadre of healthcare professionals having a clearly defined task. However, inaccuracy in task distribution and description can result in dishonesty, interpersonal disputes among healthcare professionals, and inefficiency in task distribution when providing customers with healthcare services [7]. According to Le *et al*. [1], the provision of time slots for healthcare workers to perform additional shared tasks was insufficient, resulting in lower engagement, especially for patients whose financial status restricted their ability to attend rendezvous, lowering the quality of care. Moreover, physical space limitations in health clinics presented a major obstacle to task-sharing intervention, making it difficult to achieve privacy for private counselling sessions with clients [1].

***Client characteristics:*** client characteristics are another major barrier to task shifting/sharing intervention [1]. A study conducted by Le *et al*. [1] on the barriers to adopting evidence-based task-sharing mental health interventions in low- and middle-income countries (LMICs) showed that the demographics of the clients influence their level of trust in the service being provided by CHWs involved in task sharing intervention. For instance, the more educated or higher-income clients were hesitant to receive a health service from a CHW instead of a registered nurse.

**Success indicators for task shifting and sharing:** the effectiveness of task-shifting and sharing interventions depends on a variety of indicators. These indicators are presented in Table 2.

**Table 2:** success indicators for task shifting and task sharing

	Indicators	Description/explanation
**What to task shift/share?**	Psychosocial support	Tasks can be shifted and shared from doctors to nurses and CHWs to improve psychosocial support for clients with health conditions such as HIV/AIDS, COVID-19, and obesity [15].
	Medication prescription	The prescription of certain medications can be shifted and shared between physicians and non-medical practitioners.
**Who to task shift/share?**	CHWs, Healthcare workers, Physicians, Neurosurgeons, Nurses, Midwives	Tasks can be shifted from healthcare professionals to community health workers by appropriately training community health workers and other less skilled professionals.
		Tasks can be shared between physicians’ clinicians to non-physician clinicians, including nurses, medical doctors, and neurosurgeons [16].
**How to task shift/share?**	Political constraints	Different countries have different policies, which can be very political at times. In order to avoid political constraints, the government and policymakers must agree on implementing TS/S interventions in their respective countries.
		Effective consideration of WHO's recommended guide to effectively implement TS/S in countries with political constraints.
	Funding	Different funding bodies, such as the World Bank, WHO, and UNAIDS, are required to sponsor the adoption of task shifting and sharing in LMICs.
		Other funding bodies, such as the government and non-governmental organizations, are also very important.
	Social and cultural factors	Defining and considering certain social and cultural factors influencing task-shifting implementation is critical.
		Religious norms and beliefs.
**Where to task shift/share?**	Community, hospital	Shifting and sharing tasks can be adequately carried out at the community level and hospitals/health centers [12, 14].

**The main criteria for task shifting and sharing:** several indices determine if task shifting and sharing are viable options in any given work environment. These conditions influence the decision of when and if shifting and sharing of tasks should be adopted, the intention being to arrive at success ultimately.

***Insufficient manpower:*** task shifting and sharing are strategic responses to address manpower shortages in healthcare settings. These approaches involve reallocating tasks among different cadres based on effectiveness rather than qualifications. They help maximize team outcomes despite workforce deficits, making them suitable in scenarios of inadequate manpower. In regions like Africa with marked human resource gaps, exploring task shifting and sharing becomes crucial, though they should not substitute skilled staff recruitment. Additionally, task sharing becomes pertinent when rapid response is essential due to insufficient trained personnel. The need for swift healthcare reactions driven by limited qualified workforce underscores the rationale for task shifting and sharing. This interplay between manpower scarcity, rapid response, and efficient resource allocation shapes the application of task sharing and shifting.

***Complexity of care:*** in the health sector, this necessitates the need to share different tasks. Take, for instance, in the surgical context, tasks will be shared among surgeons/clinicians who have the capacity and have been trained to perform surgery in order to boost output, address the burden of new tasks, improve access, and solves human resource shortages [9].

***Sustainability:*** this is another key factor in task delegation and sharing. In initiatives like Nigeria's Reproductive, Maternal, Newborn, and Child Health (RMNCH), community health extension workers (CHEWs) are preferred due to lower remuneration, addressing resource constraints [15]. Some countries can't afford full remuneration for higher-skilled healthcare workers, leading to task shifting and sharing as alternatives. These strategies compensate for staff shortages by employing less-skilled workers who won't demand full remuneration packages, ensuring sustainability in healthcare provision. A summary table of when we should task shift and task share is presented in Table 3.

**Table 3:** what are the main criteria for task shifting and task sharing? (when should we task shift/task share?)

Criterion	Description/explanation
**Shortage of/lack of sufficient Manpower**	Task shifting/sharing is often explored when a healthcare workforce shortfall exists.
**The necessity for a rapid response**	Task shifting/sharing is also a viable option when there is a need for a rapid response within a short time frame. For instance, core medical tasks can be shifted or shared with less qualified health workers in cases of emergency.
**Broad consultation**	Task shifting should only be employed after broad consultation with the relevant stakeholders in the particular sector.
**When there is an identified need**	Professional shortcomings identified by stakeholders (i.e., lack of neurosurgeons), such as the Ministry of Health of the relevant country.
**When there is a supportive group for task sharing/shifting**	These would be the people with the knowledge to support sharing/shifting with individuals or groups.
**When there is a person or team responsible for the work associated with the task shifting/sharing activities**	People (knowledge recipients) with the capacity to understand and execute the work and associated ethical considerations.
**When regulatory considerations are understood**	Governments, professional bodies, and ethics groups assume the responsibility to ensure that the activities support the need of the patient(s).
**When there are terms for task shifting/sharing**	How long will the shifting/sharing of tasks last?
**When there are defined training considerations**	What training will be received before task shifting/sharing of tasks commence?

**Success criteria for effective task shifting and sharing:** the advantages of task shifting and sharing may not be immediately apparent for health workers. Some criteria for evaluating the effectiveness of task shifting and sharing are shown in Table 4.

**Table 4:** success criteria for effective task shifting and task sharing?

Indicator	Description/explanation
**Improved quality of patient care**	Task shifting/sharing should ostensibly result in faster and better patient healthcare. When this is achieved, it is safe to say then that task shifting/sharing was successful.
**Cost effectiveness**	One clear sign of a successfully implemented task sharing/shifting is a reduction in the cost of providing health services to patients.
**Improved ease of access to healthcare**	Task shifting/sharing is said to have been successful when patients can access healthcare relatively easily.
**Protocols outline expectations and reports to capture outcomes**.	This will provide objective evidence of task-shifting/sharing activities. These documents would be subjectable to audits.
**Specific delegation of tasks and certification of relevant training**	Delegation should be specific where a traceability matrix can be created for who was the trainer (of the service provider), the service provider, and the outcome of each procedure.
**Part of the literature suggests that the focus should be on task sharing and less reliance on task shifting**	This would allow for capacity building toward sustainability. This is because task shifting is associated with poor outcomes from the assignee’s point of view. This suggests that more support should be given.
**Matrix on collaboration and coordinated care**	To streamline the process.
**Education**	Recruits have the necessary education to engage in task-shifting/sharing activities.

***Improved quality of patient care:*** primarily, task sharing and shifting should result in more seamless, enhanced patient care. This is due to eliminating the bottlenecks that typically bedevil the patient care system, increasing collaboration between healthcare workers, entrenching a person-centered approach to healthcare, and introducing more healthcare workers. Thus, one indicator that task shifting and sharing has successfully been carried out is an improved quality of patient care. This could be in the form of reduced wait time, increased access to counseling/care, and reduced patient mortality rate [8].

***Efficient use of resources/cost effectiveness:*** task shifting and task sharing can be motivated by economic incentives, that is, the need to use resources effectively. This is because the process sees the better utilization of available manpower and eliminates the need to outsource tasks. Furthermore, physicians can concentrate more on complicated cases thanks to task shifting, which also lowers the cost of personnel management. In light of this, a substantial decrease in the overall cost of maintaining the healthcare system is frequently a sign that task shifting and sharing are successful. In a review of 34 studies on the possible cost savings of task shifting, 30 studies revealed a drop in health costs due to the adoption of task shifting and task sharing, both to the health system and the client [13].

### Ethical considerations for task shifting and task sharing

***Justice principle:*** respect is a concept that shapes the principle of justice. The principle of justice states that everyone must be handled fairly and given the same opportunity to be listened to and considered. Justice makes sure that everyone has an equitable opportunity to participate in legal proceedings [30]. Although higher-educated healthcare worker cadres are required to provide CHWs with adequate guidance, supervision, and management, CHWs are often given limited chances to provide feedback in HIV programs [30]. As a result of institutional policies that have consistently prohibited them from taking part in HIV initiatives, this draws attention to procedural justice violations. Since CHWs may believe they have few chances to provide feedback within the delivery of HIV care, this problem may impact motivation and retention rates.

***Respect for persons:*** according to the principle of respect for persons, all persons should be respected. It is critical that healthcare professionals, including CHWs, physicians, and nurses, receive enough knowledge to exercise their full autonomy while making wise decisions. This is crucial for CHWs since they frequently find themselves in precarious situations because their level of education is low compared to other members of the medical community. Despite this, several CHWs have claimed they were enlisted for interventions without receiving the necessary instruction or knowledge about carrying out their duties [30]. It is important to give prospective CHWs detailed instructions on their responsibilities throughout the recruitment process to get around this conundrum. Additionally, they should be informed upfront if they will be paid for their labor and if there are any chances for professional progression. Each of these initiatives can help CHWs do their duties with greater knowledge.

***Beneficence:*** according to the concept of beneficence, promoting benefits to human welfare and health should be a goal of healthcare delivery [30]. This idea minimizes possible harm while promoting the welfare of individuals and communities. The welfare gains may include health gains and social benefits like neighborhood empowerment. This idea is especially pertinent to HIV initiatives. For instance, when providing HIV services, CHWs may be forced to conduct home HIV tests without the proper safety precautions, putting their health at risk. Therefore, precautions should be taken to prevent these problems, and sufficient welfare should be offered. For instance, Governments and pertinent organisations can ensure CHWs have access to the right tools, like latex sleeves, when performing HIV testing that would help CHWs maintain their health [30].

***Proportionality:*** the principle of proportionality asserts that moral considerations and public health benefits hold equal importance [30]. It dictates that positive aspects should be balanced against negatives in decision-making, vital for task shifting and sharing interventions. This principle guides assessment of options and helps decision-makers choose the least disruptive approach by considering personal gains alongside societal well-being.

***Cultural humility:*** in order to foster greater collaboration and partnership-building during task-shifting and sharing interventions, cultural humility highlights the significance of stakeholders being receptive to exchanging cultural knowledge and skills throughout healthcare delivery [30].

**Conclusion and recommendations:** task shifting and sharing are potential interventions for expanding healthcare within LMICs and increasing human resources for health. In this article, we have been able to discuss its evidence, challenges, and opportunities in sub-Saharan Africa, and as a result, we recommend that LMICs should: consider implementing, extending, and strengthening task shifting or task sharing methods in areas where health staff shortages are impeding the availability of HIV and other health services. Task shifting should be implemented alongside other initiatives aimed at increasing the number of qualified health workers [4]. Analyze a framework for researching task shifting or task sharing in order to address other important public health issues. In addition, LMICs should perform a human resource analysis to provide data on the demographics of current HRH in both the public and private sectors. Examine and think about using current regulating methods, such as laws, proclamations, rules, regulations, policies, and guidelines, to permit cadres of health workers to practice within a wider range of practice and to permit the emergence of new cadres within the health workforce. Specify the responsibilities and corresponding competency levels needed for both newly established cadres created due to the task shifting/task sharing approach and current cadres expanding their practice areas
